# Tibial stem extension versus standard configuration in primary cemented total knee arthroplasty: systematic review and meta-analysis

**DOI:** 10.1186/s13018-024-05342-2

**Published:** 2025-01-06

**Authors:** Arash Heidari, Mohammad H. Ebrahimzadeh, Mahla Daliri, Ali Moradi, Mahdieh Sahebi, Masoumeh Sadeghi

**Affiliations:** 1 Orthopedic Research Center, Ghaem Hospital, Mashhad University of Medical Sciences, Mashhad, Iran; 2 Department of Epidemiology, School of Health, Mashhad University of Medical Science, Mashhad, Iran

**Keywords:** Total knee arthroplasty, Stem extension, Standard configuration, Tibial loosening, Clinical outcome, Prosthesis

## Abstract

**Background:**

In order to increase the stability of tibial component in total knee arthroplasty (TKA), intramedullary stem extensions (SE) have been developed. The aim of this systematic review and meta-analysis is to address the critical knowledge gap on post-operative outcomes and complications rate comparison between tibial component with SE compared to the tibial component standard configuration (SC) in primary cemented TKA.

**Methods:**

We conducted a comprehensive search of online databases, including Pubmed, Embase, ISI Web of science, Cochrane Library, and Scopus, using the following MeSH terms, (total knee arthroplasty) OR (TKA) OR (total knee replacement) AND (Tibial stem) OR (stem extension) OR (long stem). We included clinical studies that compared the tibial SE with no tibial stem (standard configuration) in primary cemented TKA. The important exclusion criteria were studies on revision (secondary) TKA, un-cemented arthroplasty, high level constrained implants, TKA with tibial augment & tibial bone graft, TKA with femoral stems, studies on short tibial keel (shorter than SC), without any tibial keel, studies with less than 12 months of follow-up. Knee Society Score (KSS) functional and clinical scores were considered as clinical outcomes along with tibial loosening and implant survival rate. The retrieved studies were assessed for methodological quality using Cochrane Collaborations tool for assessing the risk of bias in randomized trials (ROB) and Cochrane Risk of Bias in Non-Randomized Studies—of Interventions (ROBINS-I) tools. Weighted mean difference (WMD) with 95% confidence interval (CI) was calculated using random-effects meta-analysis taking into account for heterogeneity.

**Results:**

A total of 223,743 patients (223,766 knees) from 15 articles were included. The risk of tibial aseptic loosening is 54% lower on average in SE group in comparison with SC group (RR: 0.46; 95% CI: 0.29 to 0.74), which is more notable among obese class I patients (RR: 0.47; 95% CI: 0.28 to 0.78), but not significantly different among obese class II patients (RR: 0.58; 95% CI: 0.19 to 1.78). KSS functional and clinical score increased 3.85 score (95% CI: 1.52 to 6.18), and 1,24 scores (95% CI: − 0.22 to 2.70) among patients in SE group, respectively. The survival rate was 1.04 times greater in the SE group. There was no notable difference in terms of knee deformity (hip-knee-ankle angle) correction, all cause secondary procedure, and complications rate between the two groups.

**Conclusion:**

The meta-analysis of post-operative functional scores and tibial loosening rate indicates a preference for tibial SE over the SC in primary cemented TKA. Some studies were rated as having a fair to critical risk of bias during the quality assessment. To strengthen the evidence and improve the applicability of our findings in clinical practice, future high-quality studies are required.

**Supplementary Information:**

The online version contains supplementary material available at 10.1186/s13018-024-05342-2.

## Introduction

Total Knee Arthroplasty (TKA) relies on the mechanical stability of the implanted femoral and tibial components. According to recent research, aseptic loosening has emerged as the primary reason for failure after TKA, surpassing infection in terms of prevalence [1, 2]. To enhance the stability, intramedullary stems, have been developed for the femoral and tibial components [3]. These stems are particularly useful in managing complex deformities and addressing the increasing burden of revision total knee arthroplasty (rTKA), in which the prosthesis stability is a challenging issue [4, 5]. Stems enhance overall stability by redistributing loads from weakened metaphysis to the diaphysis, thereby reducing strain at the interface between bone and component [6]. The use of tibial stems can reduce proximal tibial strain by 20% to 60%, depending on bone stiffness [4, 7]. Tibial stems provide several benefits, including increased resistance to shear forces, reduced tibial lift-off, and enhanced stability by minimizing micro motion [3, 8].

It is important to acknowledge that employing longer tibial stems costs more [9] and may also have certain disadvantages, specifically concerning stress shielding in proximal of tibial bone. Wolff's Law declares that bones undergo remodeling during the chronic phase as a result of the stress and strain. Thus, stress shielding can lead to a decline in proximal bone density [10], thereby theoretically raising the likelihood of complications like loosening and peri-prosthetic fracture [11, 12]. Indications of stem extensions (SE) use in rTKA is more obvious than their use in primary TKA (pTKA). In rTKA, where bone loss and instability are common, longer stems provide additional stability and compensate for compromised bone integrity [13–15]. These stems effectively address the challenges of revision cases that require enhanced fixation and load transfer [16]. However, pTKA generally involves intact bone and excludes severe bone loss and instability, and the application of a stem may increase bone loss and thus complicate if a future revision is required [17]. Nonetheless, risk factors such as obesity [18], pre-operative deformity [19, 20], and osteoporosis can disrupt stability in pTKA, and SE was associated with a lower risk of revision for aseptic loosening [21], warranting consideration SE [22].

A systematic review and meta-analysis on obese patients in 2024 reported that SE was associated with a lower risk of aseptic loosening [23]. However, as the authors also mentioned, there was a lack of high-quality literature available. Furthermore, the exclusion of studies in which the rate of aseptic loosening was reported to be zero, as well as the miss of some studies that fulfilled the inclusion criteria in their systematic search, further limits the conclusion of this review. Furthermore, some high-quality registry studies have been published recently on this topic; the inclusion of these could enhance our study findings. Thus, a comprehensive systematic review comparing SE with the standard configuration (SC) to consolidate the literature's conflicting results and draw a conclusive analysis on this topic is yet to be undertaken.

**Fig. 1:**
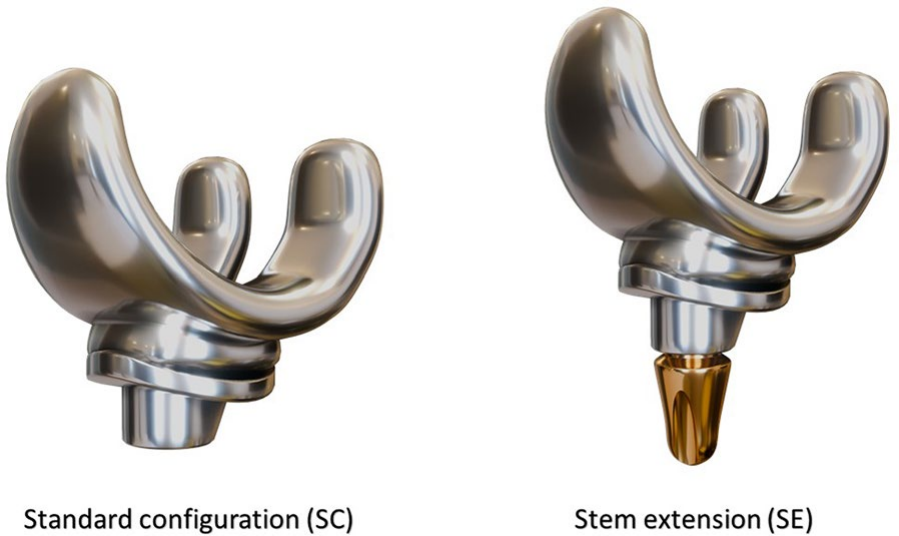
Stem extension vs Standard configuration

The aim of this article is to address the critical knowledge gap by conducting a comprehensive systematic review and meta-analysis focused on 1. post-operative outcomes and 2. complications between tibial component with SC compared to the SE, in cemented pTKA (Fig. 1).

## Methods

### Protocol

We followed the PRISMA guideline for this systematic review and meta-analysis [24]. The protocol for this study was registered in the International Prospective Register of Systematic Reviews, PROSPERO, under the registration number CRD42023443511. IRB Approval was not required.

### Search strategy

We conducted a comprehensive search of online databases, including Pubmed, Embase, ISI Web of science, Cochrane Library,and Scopus,up until March 2024.Two independent researchers (AH and MD) performed the search using MeSH terms, (total knee arthroplasty) OR (TKA) OR (total knee replacement) AND (Tibial stem) OR (stem extension) OR (long stem). We did not employ any additional filters and limited our search to English clinical trials. Additionally, we reviewed grey literature sources such as meeting abstracts and the reference lists of retrieved publications to identify any relevant studies not identified in the initial database search.

### Eligibility criteria and study selection

The question of this systematic review and meta-analysis is: What is the difference in post-operative outcomes and complication rates between tibial components with SE and those with SC in primary cemented total knee arthroplasty? To answer this question, we determined the study eligibility applying the following PICOD protocol. This involved screening studies that met the following criteria: P (Problem): primary total knee arthroplasty; I (Intervention): tibial SE; C (Comparison): tibial SC; O (Outcomes): complications and clinical outcomes; D (Design): randomized or non-randomized clinical trials. We established specific exclusion criteria of the studies focusing on joints other than the knee, revision (secondary) TKA, un-cemented arthroplasty, high level constrained implants (CCK & RHK), TKA with tibial augment & tibial bone graft, TKA with femoral stems, studies on short tibial keel (shorter than SC) [25, 26] without any tibial keel [10, 27], studies with less than 12 month of follow-up, cadaveric studies, biomechanical studies, book chapters, letters to the editor, case reports, reviews, expert comments, and studies with incomplete data.

**Fig. 2:**
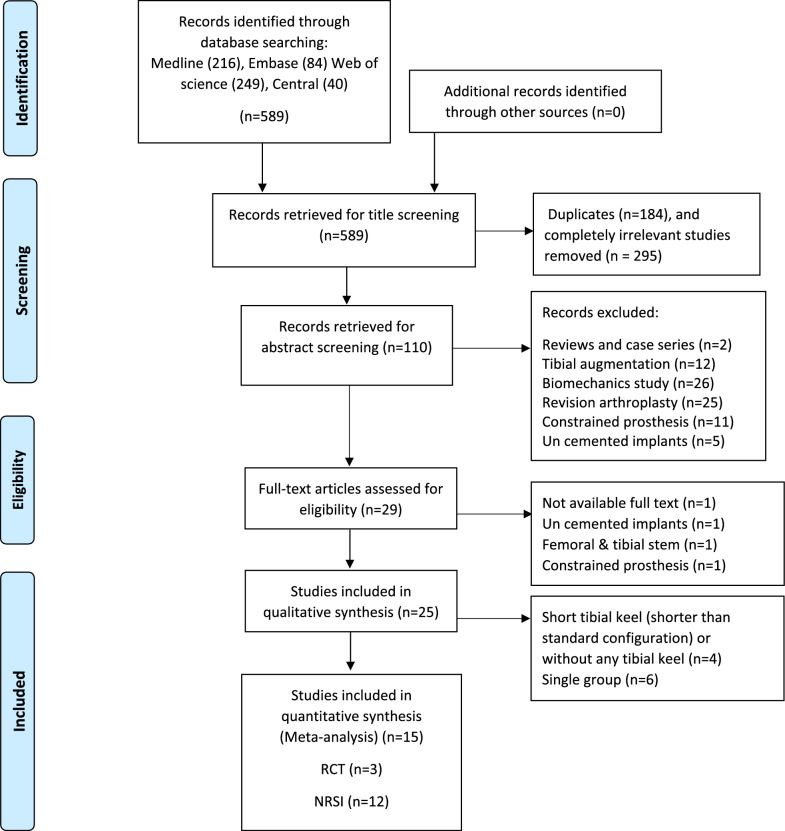
PRISMA flowchart for selection and screening of studies

Initially, 589 papers were identified for the study. Two independent researchers conducted the screening process. After removing duplicates across the databases, 110 articles remained. Following title and abstract screening based on the exclusion criteria, 29 articles were selected for full-text screening. During the full-text screening, 4 additional papers were excluded due to the use of uncemented implants, stems for both tibial and femoral components, or constrained prostheses, leaving 25 articles. Finally, 10 articles were excluded from the meta-analysis because they were either before-and-after single-group studies or involved the use of short tibial keels (shorter than the standard configuration) or no tibial keel. Ultimately, 15 articles were included in the meta-analysis. Figure 2 provides a schematic representation of the process used to identify, screen, and select studies for inclusion.

### Data extraction

Using a pre-designed Excel form, we extracted relevant information from eligible clinical trials. The extracted characteristics included the first author's name, study year, study design, population, sample size (number of knees), mean age, male-to-female ratio, BMI, follow-up duration, total tibial component (tibial keel + stem) length in each group, and prosthesis type in each group (Table 1). We also collected data on clinical outcomes, including revision rate due to tibial implant loosening, all complications caused secondary procedures, Knee Society Score (KSS) functional and clinical scores, femur-tibial angle, survival years, and complications rate, such as osteolysis (Table 2). Two researchers (AH and MD) independently extracted the data, and any discrepancies were resolved through consensus with the clinical and biostatical supervisors.

**Table 1:** Included studies’ descriptive information

N	Author (year) (ref)	Country	Study design	Population	Group	Sample size (N)	Total length (mm)	Prosthesis Constrain	Age (year)	Sex (M/F)	BMI	Mean follow-up (month)
1	Samy [30]	Egypt	NRSI	OA, RA, ACL injury, Meniscal injury	SE	99	SC + (75–100)	PS	68.1 ± 7	35/51	31.7 ± 5	69.7 ± 5
					SC	92	36–47		65.2 ± 5	34/48	28.8 ± 3	75.1 ± 2
2	Hinman [21]	USA	NRSI	–	SE	10,476	–	CR and PS	66.5 ± 9.2	3505/6971	33.4 ± 6.8	30 (median)
					SC	10,476	–		66.7 ± 8.7	3449/7027	33.3 ± 5.8	31 (median)
3	Mohammad [29]	Egypt	RCT	Primary OA	SE	130	SC + 100	PS	57 ± 2	44/86	35.56 ± 3.35	73.2 ± 8.4
					SC	134	45	PS	57 ± 4	46/88	35.15 ± 3.3	73.2 ± 8.4
4	Sathappan [36]	Singapore	NRSI	–	SE	18	SC + 75	PS	72 ± 4.3	4/14	30.9 ± 3.7	87.7 (55–180)
					SC	23	40	PS	69 ± 2.1	5/18	31.3 ± 2.3	87.7 (55–180)
5	Park [34]	Korea	NRSI	Primary OA	SE	88	SC + 30	PS	67.18 ± 5.47	1/87	27.60 ± 3.69	109.22
					SC	88	36–47	PS	67.28 ± 6.69	1/87	27.12 ± 3.66	103.81
6	Steere [37]	USA	NRSI	BMI ≥ 35	SE	50	SC + 30	PS	61 ± 8.8	7/43	44.4 ± 6.1	30 ± 3.8
					SC	128	23–47	PS	62 ± 8.7	26/102	39.8 ± 4.1	34 ± 5.2
7	Fournier [31]	France	NRSI	BMI > 30	SE	35	X + 30	PS	69.3 ± 8	26/9	34.6 ± 2.2	52 ± 21
					SC	105	X	PS	69.5 ± 7.3	85/20	34.6 ± 2	50 ± 22
8	Fournier [32]	France	NRSI	Severe varus (HKA < 170)	SE	45	X + 30	PS	70.4 ± 7.5	30/15	32.1 ± 5.7	57 ± 27
					SC	135	X	PS	72 ± 7.3	100/35	30.1 ± 4.4	64 ± 37
9	Elzohairy [27]	Egypt	RCT	BMI ≥ 35	SE	92	SC + 100	PS	55.69 ± 8.45	60/32	38.84 ± 3.89	91.2 ± 12
				degenerative or posttraumatic OA	SC	88	36–47	PS	57.3 ± 7.8	52/36	40.0 ± 3.95	91.2 ± 12
10	Parratte [28]	France	RCT	BMI = 30–35	SE	30	SC + 100	PS	67 ± 11	13/17	32 ± 1	36 ± 9.6
					SC	30	36–47		69 ± 7	12/18	33 ± 2	
				BMI ≥ 35	SE	30	SC + 100	PS	69 ± 9	13/17	39 ± 2	36 ± 9.6
					SC	30	36–47		68 ± 7	12/18	38 ± 2	
11	Garceau [33]	USA	NRSI	OA	SE	500	SC + 30	PS,CPS,US	65.4 ± 8.8	213/287	31.3 ± 5.6	42 ± 6
					SC	850	23–40	PS,CPS,US	64.7 ± 8.8	362/488	31.1 ± 5.6	60 ± 9.6
12	Garceau [35]	USA	NRSI	OA	SE	162	SC + 30	PS	65.4 ± 10.8	68/94	32.9 ± 0.6	36 ± 7.32
					SC	74	23–40	PS	64.7 ± 9.1	26/48	30.6 ± 0.7	37.2 ± 12
13	Osan [38]	Australia	NRSI	OA	SE	15,194	-	CR, PS	68.1 ± 9.2	6623/8571	33.6 ± 7.8	
					SC	117,876	-	CR, PS	69 ± 8.9	50,961/669	32.2 ± 22.8	79.2 ± 57.6
14	Osan [39]	Australia	NRSI	BMI < 30	SE	3091	X + (30–100)	CR, PS	70.4 ± 9.4	1552/1539	26.4 ± 2.6	33.6 ± 24
					SC	23,674	X		70.8 ± 8.7	11,389/123	26.5 ± 2.5	37.2 ± 22.8
				BMI ≥ 30	SE	5729	X + (30–100)	CR, PS	66 ± 8.8	2132/3597	37.5 ± 6.8	30 ± 22.8
					SC	34,014	X		67.2 ± 8.4	14,172/198	36.2 ± 29	38.4 ± 22.8
15	Druel [40]	France	NRSI	30 < BMI < 35	SE	30	SC + 30	PS	67/9 ± 8	17/13	31/7 ± 1/4	24
						30	SC + 100		69 ± 9/4	10/20	32/2 ± 1/4	24
					SC	30	36–47	PS	69/7 ± 6	6/24	32 ± 1/3	24
				BMI > 35	SE	30	SC + 30	PS	68/3 ± 6/4	11/19	39/2 ± 4/2	24
						30	SC + 100		69 ± 10/5	4/26	39/1 ± 4/2	24
					SC	30	36–47	PS	68/2 ± 5/5	4/26	37/8 ± 3	24

OA: osteoarthritis; NRSI: non-randomized study of intervention; RCT: randomized clinical trial; M/F: male/female; SE: stem extension; SC: standard configuration

**Table 2:** Included studies clinical outcomes description

N	Author (year) (Ref)	Group	Pre-op deformity (HKA angle) *	Post-op deformity (HKA angle) *	KSS Functional score (mean ± SD)	KSS Clinical score (mean ± SD)	Aseptic Tibial loosening (N)	Survival (%) 5–10 year	Total post-op complications (except Aseptic tibial loosening) (N)	Total cause revision (secondary procedure)
1	Samy [30]	SE	8 ± 3.11	0 ± 2.1	90.7 ± 1.5	95.3 ± 2.8	2	–	0	2
		SC	4 ± 2.8	0 ± 1.9	88.2 ± 6.2	94.6 ± 3.6	2	–	0	2
2	Hinman [21]	SE	–	–	–	–	15	–		-
		SC	–	–	–	–	33	–		-
3	Mohammad [29]	SE	9 ± 2.9	- 5 ± 3.5	74.8 ± 5	92.5 ± 2	0	100	1(intra operative FX)	0
		SC	8.2 ± 3	- 5.1 ± 3.2	73.4 ± 5.1	92 ± 2	0	100	1 (infection)	1
4	Sathappan [36]	SE	13.4 (8–35)	2.9 ± 1.3	–	–	0	–		-
		SC	13.4 (8–35)	2.1 ± 0.8	–	–	0	–		-
5	Park [34]	SE	16.08 ± 4.62	2.86 ± 2.95	–	–	0	100		-
		SC	15.90 ± 4.54	2.57 ± 3.30	–	–	5	97.5		-
6	Steere [37]	SE	–	–	–	–	0	–	4	4
		SC	–	–	–	–	0	–	6	6
7	Fournier [31]	SE	5.7 ± 4.9	4.5 ± 4.1	73 ± 22.8	83 ± 15	0	100	5	5
		SC	3.8 ± 5.2	1.7 ± 3.8	77 ± 20.76	86 ± 14.7	7	85	4	11
8	Fournier [32]	SE	13.8 ± 4	5 ± 5	77.2 ± 19	84.9 ± 14	0	100	4	4
		SC	12.9 ± 3	3 ± 3.2	74.8 ± 21	87.3 ± 11	4	96	8	12
9	Elzohairy [27]	SE	–	4.7 ± 6.58	77.61 ± 13.53	–	3	–	4	7
		SC	–	4.1 ± 5.98	67.76 ± 16.5	–	5	–	19	24
10	Parratte [28]	SE	0 ± 2	–	75 ± 7	84 ± 5	0	–	2	1
		SC	0 ± 2	–	72 ± 6	82 ± 5	0	–	3	0
		SE	1 ± 2	–	74 ± 6	85 ± 6	0	–	4	0
		SC	1 ± 2	–	66 ± 5	78 ± 6	1	–	4	1
11	Garceau [33]	SE	–	–	–	–	0	100		-
		SC	–	–	–	–	12	98.5		-
12	Garceau [35]	SE	–	–	–	–	0	100		-
		SC	–	–	–	–	4	94.5		-
13	Osan [38]	SE					HR (95% CI) = 0.23 (0.11–0.50)			405
		SC								3433
14	Osan [39]	SE BMI < 30								54
		SC BMI < 30								
		SE BMI ≥ 35								110
		SC BMI ≥ 35								
15	Druel [40]	SE 30			90.4 ± 6.3	88.9 ± 8.1	1	90 (2 year)	0	1
		SE 100			78.4 ± 9.1	79 ± 8.4	0	100 (2 year)	0	0
		SC			68.5 ± 17	80.6 ± 13.7	0	100 (2 year)	1	1
		SE 30			85.5 ± 7.8	84.5 ± 8.1	0	100 (2 year)	0	0
		SE 100			73 ± 9.7	72.4 ± 9.6	0	90 (2 year)	2	2
		SC			61.8 ± 12	78.2 ± 9.5	1	100 (2 year)	0	1

SE: stem extension; SC: standard configuration; HKA angle: Hip-Knee-Ankle angle; KSS: knee society score

^*^The positive data are in varus, and negative data are in valgus

### Quality assessment

**Fig. 3:**
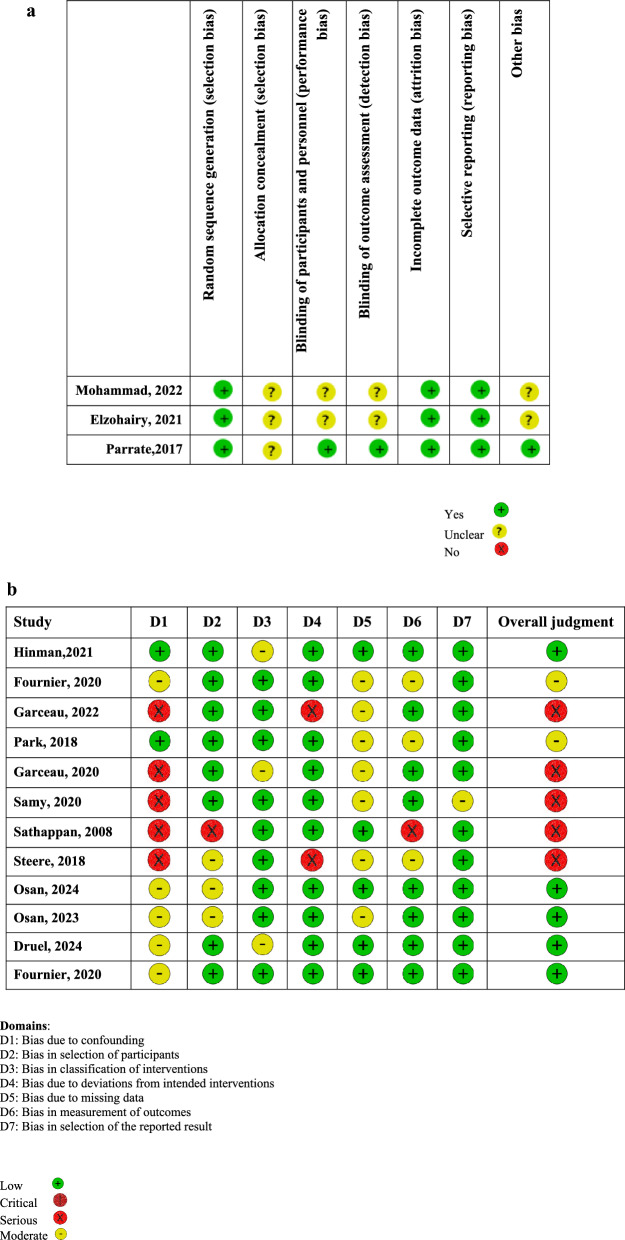
Risk of Bias Summary (ROB tool) (A); Risk of Bias Summary (ROBINS-I) (B)

Assessment of the methodological quality of the included controlled clinical trials (RCTs) was conducted by the Cochrane Collaborations tool for assessing the risk of bias in randomized trials (ROB) (Fig. 3A). This tool is used to evaluate seven domains of bias: random sequence generation (selection bias), allocation concealment (selection bias), blinding of participants and personnel (performance bias), blinding of outcome assessment (detection bias), incomplete outcome data (attrition bias), selective reporting (reporting bias), and other sources of bias (other bias). A risk of bias judgment was made for the domain as either “low risk of bias”, “unclear risk of bias”, or “high risk of bias”. An overall judgment of the risk of bias is then provided for each study. Two reviewers (AH and MD) assessed the risk of bias, and a third reviewer (MS) verified the assessments.

For non-randomized studies of intervention (NRSI), we employed the Cochrane Risk of Bias in Non-Randomized Studies—of Interventions (ROBINS-I) tool (Fig. 2B), a validated tool for assessing the quality of non-randomized studies. This tool assesses the risk of bias across multiple domains, including confounding, participant selection, classification of interventions, deviations from intended interventions, missing data, outcome measurements, and selective reporting. Each domain is evaluated for the risk of bias, categorized as low, moderate, serious, or critical.

### Data analysis

The primary outcome of our meta-analysis involved comparing tibial implant loosening revision rate, as well as KSS functional and clinical scores between SC group and SE group in pTKA. Other outcomes included hip-knee-ankle (HKA) angle, implant survival, and complications rate. Forest plots were used to assess heterogeneity and calculate pooled weighted mean differences with corresponding 95% confidence intervals (WMD with 95% CI) for efficacy outcomes, and pooled percentages with 95% CI for complications. To account for the heterogeneity of study populations, a random-effects meta-analysis was conducted. Pooled estimates and their corresponding 95% CIs were calculated using inverse-variance weights. Heterogeneity across studies was assessed using I^2^ statistics, with values of I^2^ = 0% indicating no observed heterogeneity and I^2^ ≥ 50% indicating substantial heterogeneity. Cochran's Q statistic was used to analyze the statistical significance of heterogeneity. We performed sensitivity analysis to determine the impact of individual studies on heterogeneity and assess the robustness of pooled estimates. Subgroup analyses were conducted based on study design. All statistical tests were two-tailed, and a significance level of less than 0.05 was set for all analyses, except for the heterogeneity test. We conducted all statistical analyses using Stata version 17.0 (Stata Corp., College Station, TX, USA).

## Results

### Studies’ characteristics

We initially identified 589 articles from the databases, out of which 25 were deemed relevant. However, 10 articles were excluded due to insufficient quantitative data for analysis, resulting in 15 studies, comprising 3 RCTs [28–30] and 12 NRSIs [21, 31–41], for inclusion in our meta-analysis (Fig. 3).

A total of 223,743 patients (223,766 knees) were included, with 95,110 patients (43%) being male and 128,633 patients (57%) being female. The SC group included 187,867 patients with a mean age of 68.73 years, while the SE group included 35,876 patients with a mean age of 67.53 years.

### Quality assessment

All RCTs demonstrated a low risk of bias concerning sequence generation, incomplete outcome data, and selective reporting. However, all studies exhibited an unclear risk of bias regarding allocation concealment. Among the three studies, two showed unclear risk of biases related to blinding of participants, personnel, and outcome assessors (Fig. 3A). The quality assessment of NRSIs revealed that five studies (42%) had serious methodological problems. Additionally, two studies presented a moderate risk of bias and five studies had a low risk. The majority of studies showed low risk of bias in domain 3 (bias in classification of interventions) and domain 7 (bias in selection of the reported result). The primary source of bias in most studies was confounding factors (Fig. 3B).

### Meta-analysis

**Fig. 4:**
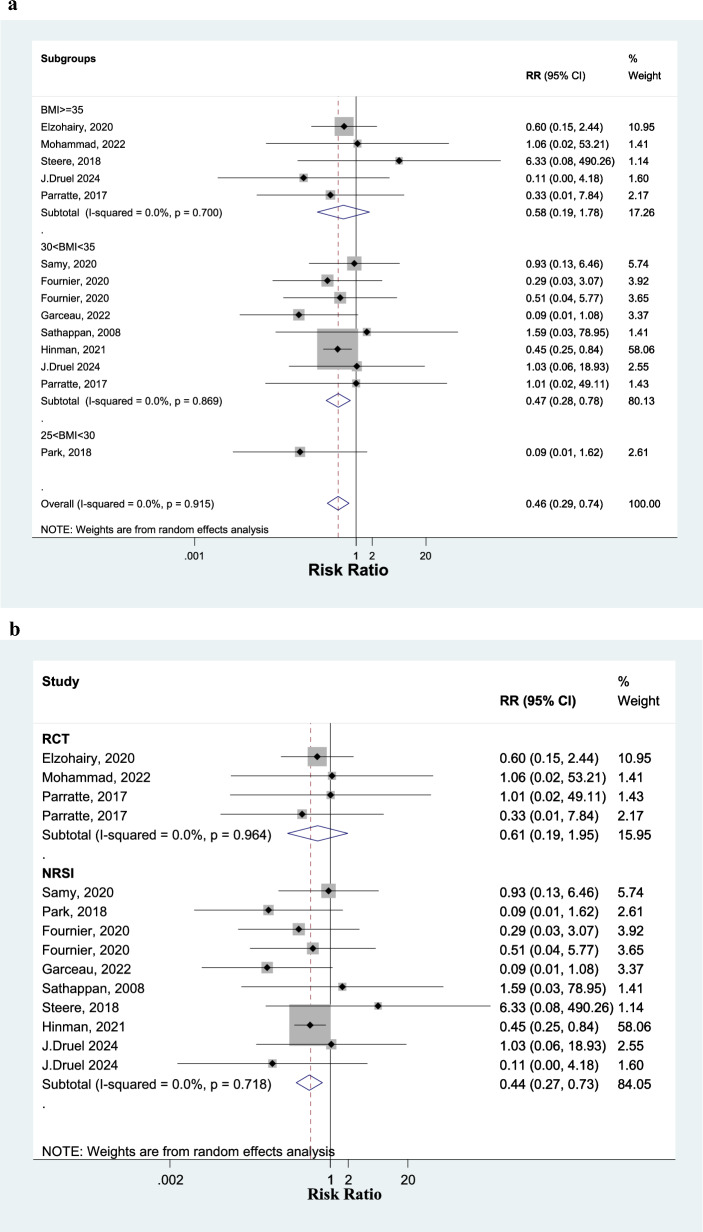
Forest plot, comparing risk of tibial aseptic loosening between tibial stem extension and standard configuration groups. Diamond represents the relative risk (pooled RR) estimate and its width shows corresponding 95% CI with random effects estimate. The size of the square and its central point reflects the study specific statistical weight (inverse of variance) and point estimate of the RR and horizontal line reflects corresponding 95% CI of the study. I2 test and Cochran’s Q statistic were used to assessing the statistical heterogeneity (P < 0.10) across studies. Subgroup analysis by BMI (A), and study design (B)
  Subgroup analysis of the three RCTs showed that the RR of tibial stem loosening is 0.61 (0.19 to 1.95) in the TKA SE group in comparison with SC group. Analyzing nine NRSIs, the RR of tibial stem loosening is 0.44 (0.27 to 0.73) in SE group in comparison with SC group (Fig. 4B).
- All cause secondary procedure
  Analyzing eight studies, the RR of all cause secondary procedure was not different between the two groups, with the WMD of 0.92 (0.83 to 1.02) in SE group in comparison with SC group (Fig. 5). Among obese class II patients (BMI ≥ 35), all cause secondary procedure is 1.12 (0.47 to 2.67) in the TKA SE group, in comparison with SC group. Among obese class I patients (30 < BMI < 35), all cause secondary procedure is 0.92 (0.83 to 1.02) in the TKA SE group, in comparison with SC group (Fig. 5).

- Tibial aseptic loosening lead to revision
  Analyzing 11 studies, the RR of tibial stem aseptic loosening is 0.46 (0.29 to 0.74) in the TKA SE group, in comparison with SC group (Fig. 4). Among obese class II patients (BMI ≥ 35), tibial aseptic loosening is 0.58 (0.19 to 1.78) in the TKA SE group, in comparison with SC group (Fig. 4A). Among obese class I patients (30 < BMI < 35), tibial aseptic loosening is 0.47 (0.28 to 0.78) in the TKA SE group, in comparison with SC group (Fig. 4A).

**Fig. 5:**
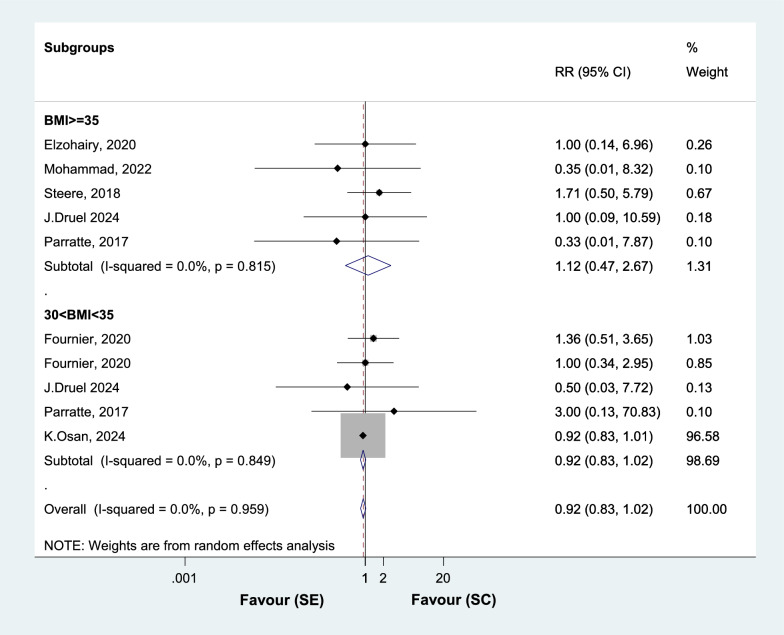
Forest plot, comparing risk of all cause secondary procedure between tibial stem extension and standard configuration groups. Diamond represents the relative risk (pooled RR) estimate and its width shows corresponding 95% CI with random effects estimate. The size of the square and its central point reflects the study specific statistical weight (inverse of variance) and point estimate of the RR and horizontal line reflects corresponding 95% CI of the study. I2 test and Cochran’s Q statistic were used to assessing the statistical heterogeneity (P < 0.10) across studies. (subgroup analysis by BMI)
- KSS Functional Score
  Analyzing six studies, KSS functional score is in average 3.85 score (95% CI: 1.52 to 6.18) higher in SE group (Fig. 6A). Subgroup analysis of the three RCTs shows KSS functional score is in average 5.34 score (95% CI: 1.22 to 9.47) higher in SE group (Fig. 6B).

**Fig. 6:**
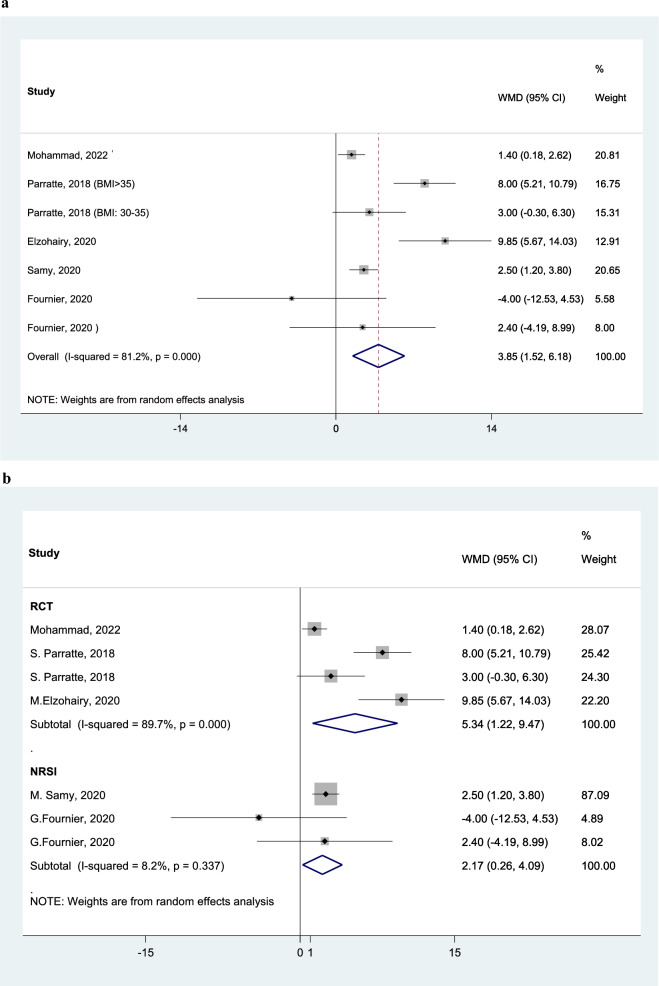
Forest plot, comparing the weighted mean difference of KSS functional score between tibial stem extension and standard configuration groups (A). Subgroup analysis by study design (B). Diamond represents the weighted mean difference (pooled WMD) estimate and its width shows corresponding 95% CI with random effects estimate. The size of the square and its central point reflects the study specific statistical weight (inverse of variance) and point estimate of the WMD and horizontal line reflects corresponding 95% CI of the study. I2 test and Cochran’s Q statistic were used to assessing the statistical heterogeneity (P < 0.10) across studies
- KSS Clinical Score
  Analyzing five studies, KSS clinical score is in average 1.24 scores (95% CI: − 0.22 to 2.70) higher in SE group (Fig. 7A). Subgroup analysis of the two RCTs shows KSS clinical score is in average 2.92 score (95% CI: − 0.59 to 6.43) higher in SE group (Fig. 7B).

**Fig. 7:**
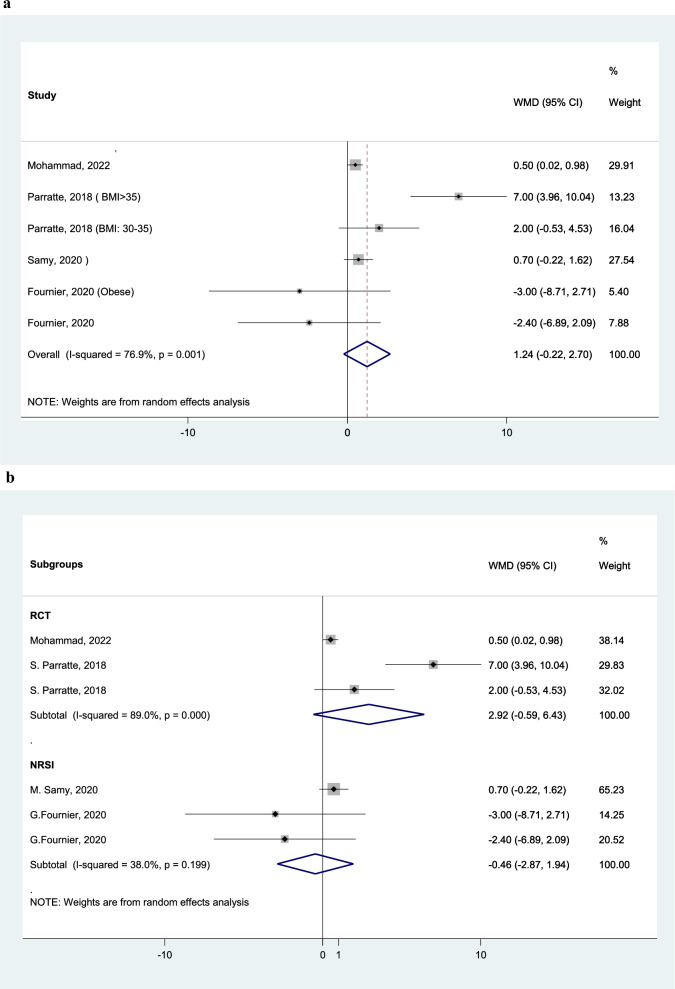
Forest plot, comparing the weighted mean difference of KSS clinical score between tibial stem extension and standard configuration groups (A). Subgroup analysis by study design (B). Diamond represents the weighted mean difference (pooled WMD) estimate and its width shows corresponding 95% CI with random effects estimate. The size of the square and its central point reflects the study specific statistical weight (inverse of variance) and point estimate of the WMD and horizontal line reflects corresponding 95% CI of the study. I2 test and Cochran’s Q statistic were used to assessing the statistical heterogeneity (P < 0.10) across studies. between stem extension and standard stem groups, separated by study design
- Prosthesis survival rate
  The prosthesis survival rate after 5–10 years is 1.04 times (1.01 to 1.07) higher in average in SE group (Fig. 8). In SE group, all the included studies reported 100% survival rate.

**Fig. 8:**
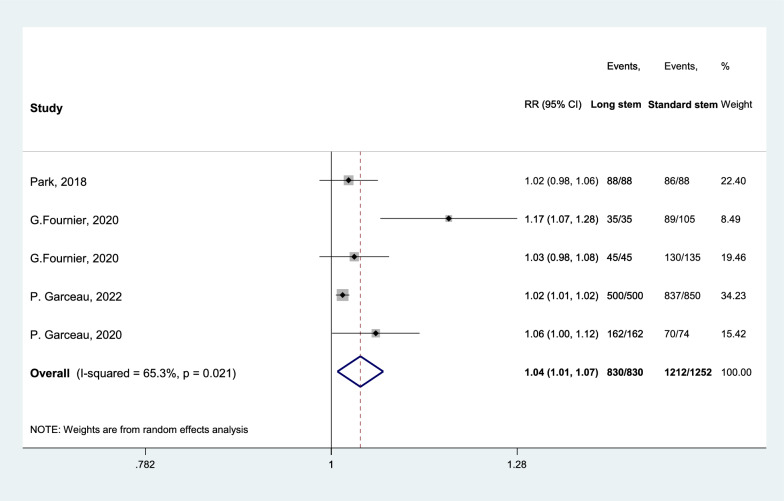
Forest plot, comparing prosthesis survival rate (5–10 Years) between tibial stem extension and standard configuration groups. Diamond represents the relative risk (pooled RR) estimate and its width shows corresponding 95% CI with random effects estimate. The size of the square and its central point reflects the study specific statistical weight (inverse of variance) and point estimate of the RR and horizontal line reflects corresponding 95% CI of the study. I2 test and Cochran’s Q statistic were used to assessing the statistical heterogeneity (P < 0.10) across studies
- Post-operative Knee deformity (HKA angle)
  Analyzing six studies, HKA angle is corrected in average 0.45 degree (95% CI: − 1.91 to 1.02) more in SE group (Fig. 9A). In the subgroup analysis among patients with pre-op deformity between 5.7 to 8 degrees varus, HKA angle is corrected in average 1.27 degree (95% CI: − 4.09 to 1.56) more in SE group (Fig. 9B).

**Fig. 9:**
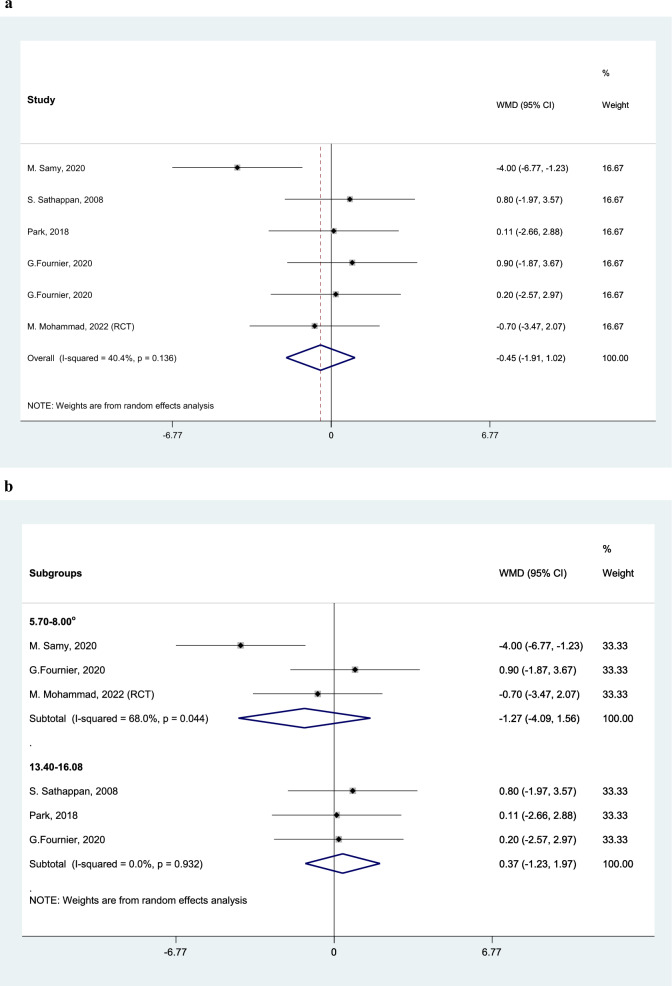
Forest plot, comparing the weighted mean difference of knee deformity (hip-knee-ankle angle) between tibial stem extension and standard configuration groups (A). Subgroup analysis by pre-op deformity angle (B). Diamond represents the weighted mean difference (pooled WMD) estimate and its width shows corresponding 95% CI with random effects estimate. The size of the square and its central point reflects the study specific statistical weight (inverse of variance) and point estimate of the WMD and horizontal line reflects corresponding 95% CI of the study. I2 test and Cochran’s Q statistic were used to assessing the statistical heterogeneity (P < 0.10) across studies
- Total complications rate (not including tibial loosening)
  Analyzing eight studies, the RR of total complications such as superficial infection, intra operative fracture was not different between the two groups, with the WMD of 0.88 (0.40 to 1.93) in SE group in comparison with SC group. I square showed moderate heterogeneity (I2: 45.9%, P = 0.055) among the reported data for complications rate (Fig. 10A).

**Fig. 10:**
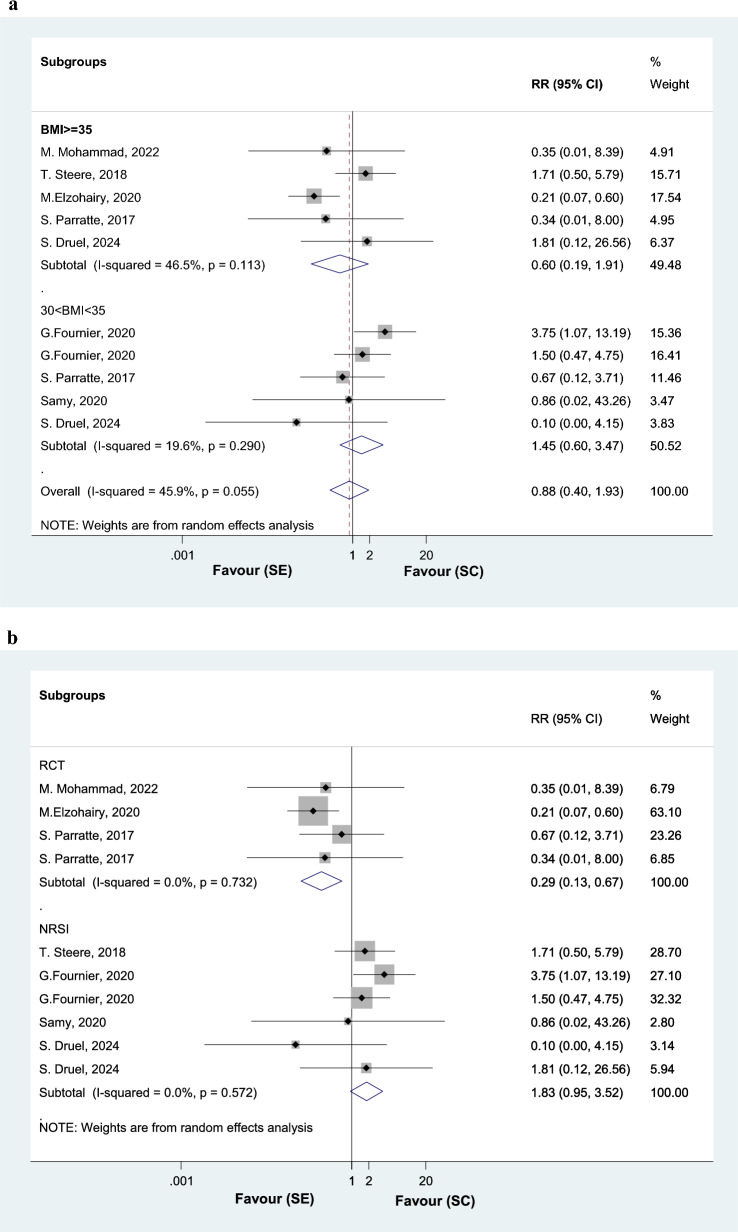
Forest plot, comparing risk of total complications’ rate (except tibial loosening) between tibial stem extension and standard configuration groups. Diamond represents the relative risk (pooled RR) estimate and its width shows corresponding 95% CI with random effects estimate. The size of the square and its central point reflects the study specific statistical weight (inverse of variance) and point estimate of the RR and horizontal line reflects corresponding 95% CI of the study. I2 test and Cochran’s Q statistic were used to assessing the statistical heterogeneity (P < 0.10) across studies. Subgroup analysis by BMI (A), and study design (B)
  Subgroup analysis of the three RCTs showed that the RR of total complications’ rate is 0.29 (0.13 to 0.67) in the TKA SE group in comparison with SC group. Analyzing five NRSIs, the RR of total complications’ rate is 1.83 (0.95 to 3.52) in SE group in comparison with SC group (Fig. 10B).

## Discussion

While surgeons have the option to apply longer tibial stems in patients considered at a higher risk for aseptic loosening, such as in rTKA, there is a lack of definitive evidence-based guidelines supporting their usage in pTKA. Our analysis indicates that primary cemented TKA procedures with tibial SE reduced risk of revision due to tibial loosening and resulted in higher KSS functional scores and prosthesis survival rate, in comparison with tibial SC.

To ensure a steady fixation, the tibial components need to effectively handle various forces such as shear, compression, and rotation at the connection point between the bone and the prosthesis [42]. The purpose of designing tibial stems is to minimize stresses at the interface between the implant and the bone. This is particularly crucial in situations where the host bone's ability to withstand these forces might be compromised [43]. Biomechanical investigations have provided evidence indicating that the implementation of tibial component stems can effectively mitigate the strain experienced in the proximal tibia [4]. A study conducted by Lonner revealed a reduction in bone density within the proximal tibia when using a tibial component with a stem, thus supports the notion that such stems play a role in alleviating proximal tibial stress [10]. On the other hand, another research conducted by Stern demonstrated that the addition of longer modular extension stems at the base of the tibial component had the consequence of increasing micromotion at the interface between the bone and the prosthesis, but the difference was less notable in cemented implants [44]. The researchers hypothesized that this phenomenon could be attributed to the extended stems engaging with the cortical bone in the diaphyseal region, resulting in a toggling effect of the tibial tray during mechanical loading at the bone-implant interface. Consequently, these findings shed light on the complex biomechanical interactions that occur in tibial components and emphasize the significance of stem design considerations for optimizing stability and stress distribution in TKA.

Osan et al. recently performed two studies on Australian Orthopaedic Association National Joint Replacement Registry data, which are ideal due to the routine capture of outcomes for different prosthesis types and provide a comprehensive view of the impact of tibial SE in pTKA, particularly in obese patients [39, 40]. Their findings suggest that while SE in TKA may offer long-term benefits, such as reducing aseptic loosening and tibial component-only revisions, they also present higher initial risks, particularly related to infection and minor revisions in obese patients. The results indicate that the use of tibial stems should be carefully considered, especially in obese patients, and highlight the need for further research to optimize patient selection and surgical techniques. Another study by Druel et al. investigated the impact of using short, long, or no tibial stems in primary TKA for obese patients, concluded that the short, cemented tibial stem offered better functional outcomes without increasing failure rates compared to a longer stem, and did not lead to increased stress in the subchondral bone. Thus, a short tibial stem may be preferable in obese patients undergoing primary TKA [41]. The high heterogeneity among the reported KSS functional and clinical scores among the studies included in our meta-analysis, could be attributed to unmeasured confounding factors (activity levels) [45], and factors related to the quality of cementing technique employed by each surgeon which have role in implant survivorship and patient satisfaction.

SE is a crucial factor in TKA, impacting strain distribution and stress shielding at the bone-implant interface. Research involving strain gauge experiments with varying metal stem lengths (20 mm, 40 mm, and 60 mm) indicated that the 60 mm stem effectively conveyed strain to the cortex, leading to reduced proximal strain. However, this effect was absent in shorter stems or all-polyethylene (PE) stems due to their reduced stiffness [46]. Present-day TKA implants commonly incorporate a central tibial keel [47], typically ranging from 35 to 50 mm, which has shown favorable survivorship with minimal or delayed clinically relevant stress shielding. The concept of stem engagement with the bone emerges as a critical determinant of its impact, surpassing absolute stem length. Classification into metaphyseal-engaging stems (MES) and diaphyseal-engaging stems (DES) is more informative. MES are typically cemented to reduce micromotion in regions with wider and more cancellous bone, whereas DES are usually press-fit, suitable for regions with narrower cortical bone. Historically, short cemented stems (30–75 mm) have been preferred for revision fixation, with longer stems for femoral fixation and shorter ones for tibial fixation. DES typically extend at least 75 mm in length [48].

Various factors, both related to the patient and the implant, can contribute to the risk of aseptic loosening, which includes obesity and the utilization of high-viscosity bone cement [49, 50]. In a study conducted by Park discovered that patients who lacked a tibial stem exhibited a higher incidence of aseptic loosening and experienced poorer overall survival compared to a group of 88 patients with tibial stems, carefully matched using propensity scores [35]. It is important to note that their study focused solely on patients with severely varus knees, whereas our study did not take into account information regarding knee alignment or deformity. Research employing Roentgen stereophotogrammetric analysis (RSA) has provided evidence of a noteworthy correlation between migration and varus alignment, as well as an augmented knee adduction moment during gait [51, 52].

Our systematic review has certain limitations that should be considered when interpreting the findings. One such limitation is related to the relatively low overall incidence of revision due to aseptic loosening observed in the included studies. While we were able to identify statistically significant differences, the limited number of events hindered our ability to perform subgroup analyses in certain patient groups considered "higher risk" for aseptic loosening, such as, younger patients or patients with severe pre-operative deformity. Consequently, this might potentially restrict the generalizability of our findings and calls for cautious interpretation. Some NRSI studies were rated as having a fair to critical risk of bias during the quality assessment. Our analysis did not account for the heterogeneity introduced by differences in implant types and variations in surgeon expertise across the included studies. Additionally, most studies did not report outcomes stratified by patient BMI, which could also contribute to heterogeneity.

## Conclusion

The meta-analysis of post-operative functional scores and tibial loosening rates indicates a preference for tibial SE over the SC in primary cemented TKA. To strengthen the evidence base and improve the applicability of our findings in clinical practice, future high-quality studies are required.

## Supplementary Information

Supplementary file 1.

Supplementary file 2.

Supplementary file 3.

## Data Availability

No datasets were generated or analysed during the current study.

## Abbreviations

TKA: Total knee arthroplasty
SE: Stem extensions
SC: Standard configuration
rTKA: Revision total knee arthroplasty
pTKA: Primary TKA
